# Effects of Chronic Roundup Exposure on Medaka Larvae

**DOI:** 10.3390/jox13030032

**Published:** 2023-09-14

**Authors:** Deborah Killian, Mehwish Faheem, Beh Reh, Xuegeng Wang, Ramji Kumar Bhandari

**Affiliations:** 1 Department of Biology, University of North Carolina Greensboro, Greensboro, NC 27412, USAwangxuegeng@scnu.edu.cn (X.W.); 2 Division of Biological Sciences, University of Missouri, Columbia, MO 65211, USA; 3 Institute of Modern Aquaculture Science and Engineering, College of Life Sciences, South China Normal University, Guangzhou 510631, China

**Keywords:** roundup, glyphosate acid equivalent, fish, embryos, development, thyroid hormone receptor

## Abstract

The use of glyphosate-based herbicides is increasing yearly to keep up with the growing demands of the agriculture world. Although glyphosate-based herbicides target the enzymatic pathway in plants, the effects on the endocrine systems of vertebrate organisms, mainly fish, are widely unknown. Many studies with glyphosate used high-exposure concentrations (mg/L), and the effect of environmentally relevant or lower concentrations has not been clearly understood. Therefore, the present study examined the effects of very low, environmentally relevant, and high concentrations of glyphosate exposure on embryo development and the thyroid system of Japanese medaka (*Oryzias latipes*). The Hd-rR medaka embryos were exposed to Roundup containing 0.05, 0.5, 5, 10, and 20 mg/L glyphosate (glyphosate acid equivalent) from the 8 h post-fertilization stage through the 14-day post-fertilization stage. Phenotypes observed include delayed hatching, increased developmental deformities, abnormal growth, and embryo mortality. The lowest concentration of glyphosate (0.05 mg/L) and the highest concentration (20 mg/L) induced similar phenotypes in embryos and fry. A significant decrease in mRNA levels for acetylcholinesterase (*ache*) and thyroid hormone receptor alpha (*thrα*) was found in the fry exposed to 0.05 mg/L and 20 mg/L glyphosate. The present results demonstrated that exposure to glyphosate formulation, at a concentration of 0.05 mg/L, can affect the early development of medaka larvae and the thyroid pathway, suggesting a link between thyroid functional changes and developmental alteration; they also showed that glyphosate can be toxic to fish at this concentration.

## 1. Introduction

Glyphosate [N-(phosphonomethyl) glycine] is an organophosphate compound used as an herbicide created by Monsanto and marketed as Roundup^®^. The herbicidal properties of glyphosate-based herbicides (GBHs) are attributed to the inhibition of 5-enolpyruvylshikimate-3-phosphate synthase (EPSPS) enzyme in the shikimic acid pathway. EPSPS plays a role in metabolic processes necessary for the synthesis of aromatic amino acids [1]. The shikimic acid pathway is not present in vertebrates [2,3] therefore, it was believed that the use of glyphosate-based herbicides will not be harmful to vertebrates, including humans. This led to the increased use of glyphosate-based herbicides in agriculture and homes. Humans and other vertebrates are exposed to glyphosate-based herbicides through dermal exposure, inhalation, ingestion of food containing glyphosate, and exposure to runoff [4,5,6]. Glyphosate is reported in natural water bodies in the range of 0.00003–0.073 mg/L. Agricultural drains have glyphosate levels ranging from 50 to 500 µg/L [5,7]. However, accidental spills and direct application resulted in higher concentrations (1.7–5.2 mg/L) of glyphosate [5]. Glyphosate is highly water soluble, and its detection in water bodies has led researchers to study the effects on non-targeted species, especially fish.

Previous literature has reported the detrimental effects of glyphosate and Roundup in non-targeted species. Exposure of zebrafish to environmentally relevant concentrations of glyphosate resulted in high levels of neurotransmitters like dopamine and norepinephrine, resulting in altered neurobehavioral response [8]. Wood frog *(Lithobates sylvaticus)* tadpoles exposed to glyphosate-based herbicide, Visionmax, resulted in decreased survival and metamorphic alterations [9]. Similarly, North American frogs, when exposed to a mixture of glyphosate-based herbicides, resulted in an increased time to reach metamorphic climax and decreased tail and snout length. The expression of thyroid hormone beta-receptor was also disrupted [10], indicating that metamorphic abnormalities may be linked with disrupted thyroid signaling. Prenatal exposure of male rats to Roundup Transorb (Monsanto) caused decreased expression of deiodinases (*dio2* and *dio3*) and thyroid hormone transporters (*Slco1c1* and *Slc16a2*) in the hypothalamus and an increased expression of *dio2*, thyroid hormone receptor genes (*thra1* and *thrb1*), and *Slc16a2* in the pituitary gland [11] further strengthening the evidence that exposure of glyphosate-based herbicides alters the thyroid signaling. Feeding abnormalities, developmental deformities, and reproductive dysfunctions are also reported in fish after exposure to glyphosate-based herbicides [12,13,14,15]. Previous studies conducted by our laboratory indicated that exposure to the environmentally relevant and high concentration of glyphosate and Roundup caused developmental and reproductive abnormalities and epigenetic modifications in medaka [16]. To further confirm the detrimental effects of glyphosate-based herbicides, the present study was designed to investigate the effects of continuous exposure to glyphosate, at concentrations lower than environmentally relevant, during the embryonic stages and evaluate its effects on development and thyroid signaling in fish.

Medaka fish was used as an animal model. Medaka fish are easy to maintain, have a maturation time of 100–120 days, eggs and embryos of medaka are transparent, and the genome is fully annotated and comparable to humans, thus making it an excellent model to study the embryonic effects of hazardous chemicals [17].

## 2. Materials and Methods

### 2.1. Fish Maintenance

The fish were reared following guidelines in accordance with the Institutional Animal Care and Use Committee (IACUC) and the UNCG guidelines for the humane treatment of test organisms during culture and experimentation. The experimental protocol was modified from Smith et al. [16] 2019. Hd-rR strain of the wild-type medaka fish (*Oryzias latipes*) was reared from an inbred line. Fish were kept at 25 ± 1 °C in 20-L water tanks. The water conditions include pH of 6.8 to 7.2, total alkalinity of 0–40 ppm, nitrate at 0–20 ppm, and ammonia at 0–0.25 ppm were maintained for fish. The tanks held constant aeration and water flow of 76 L/day. The fish tanks were under a light with a dark cycle of 14 h: 10 h. The fish were fed twice daily with granular food developed by Reed Mariculture (Campbell, CA, USA).

### 2.2. Chemical Exposure and Sampling

Three pairs of adult medaka fish (100–120 days) were placed in breeding pairs to allow natural fertilization. Eggs were collected by hand and processed to remove clumping factors and placed in embryo solution with 0.05% methylene blue. Approximately 8 h post fertilization (hpf). Ten embryos were placed each in 12 well plate containing 10 mL exposure solution. The dosing solution was prepared by dissolving the Roundup formulation in deionized (DI) water containing 0.03% sea salt. The exposure concentrations included 0 mg/L, 0.05 mg/L, 0.5 mg/L, 5 mg/L, 10 mg/L, and 20 mg/L glyphosate acid equivalent in Roundup formulation. The experiment was performed in quadruplicate. The exposure solutions were prepared in the lab using Roundup (Ready to Use) and DI water. The Roundup Ready to Use (Monsanto Corporation, Saint Louis, MO, USA) was purchased from a local vendor (Home Depot). Ready to use Roundup contain isopropylamine salt (IPA) of glyphosate as an active ingredient. The exposure continued until 14 days post-fertilization (dpf). The exposure solution changed at least 4 times during the exposure, and the methylene blue was removed from the embryo solution once fry hatched. At 14 dpf, all the fry were euthanized using MS-220 (250 mg/L) and flash frozen with liquid nitrogen. All samples were stored at −80 °C until molecular analysis was performed.

### 2.3. Phenotype Characterization

Phenotypic variations in the control and exposed groups were examined using a stereomicroscope equipped with a Nikon SMZ1000 camera and a PlanApo objective. The observed phenotypes included pericardial edema, swim bladder inflation, blood spots, pigmentation, spinal curvature, eyes defect, visceral edema, body size, and yolk coloration. The abnormalities were pictured using a Moticam 2300—3 megapixel CMOS camera under the Images Plus capture package. Hatching time and embryo survival rate was also monitored and recorded throughout the experiment.

### 2.4. qRT-PCR

The 14 dpf fry samples were homogenized individually using a hand-held homogenizer. DNA/RNA was extracted from 5 fry per treatment per replicate using the Zymo Research Duet RNA/DNA Miniprep Kit (Catalog# D7003T). RNA from each fry was considered as single biological replicate. Purity of RNA was determined using Thermo Fisher Scientific NanoDrop 2000/2000c Spectrophotometer. The RNA was reversed transcribed into cDNA using the High-Capacity Reverse Transcription Kit (Applied Biosystems). Expression patterns of genes were determined by real-time qPCR using power-Up SYBR Green Master mix (Thermo Fisher Scientific, St. Louis, MO, USA). Three technical replicates were used for each biological replicate in qPCR studies. Primers used in the present study are listed in Supplementary Table S1.

### 2.5. Statistical Analysis

All data is represented as mean ± standard error of the mean (SEM). For developmental abnormalities, data is statistically analyzed using a two-tailed *t*-test against control. Gene expression data were statistically analyzed by one-way ANOVA followed by Tukey’s post hoc test.

## 3. Results

### 3.1. Embryo Survival and Hatching Success

**Figure 1:**
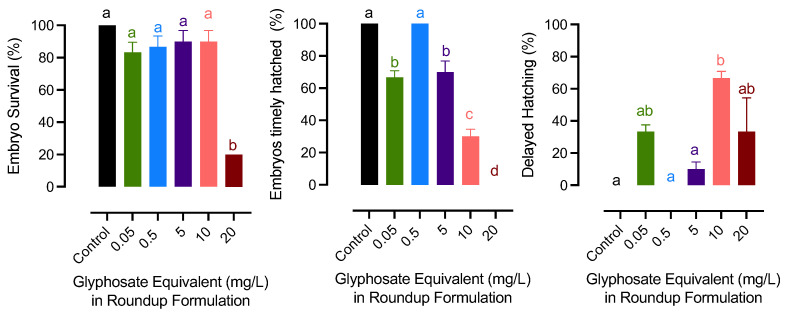
Effects of exposure to glyphosate exposure in Roundup formulation on embryo survival and hatching success in medaka. Bars with different letters on them indicate that treatments groups they represent are significantly different (*p* < 0.05) from each other. Data represent mean with a standard error of the mean.

The results have been provided as Roundup exposure (mg/L) according the reviewer’s suggestion. The mg/L was the concentration of glyphosate in the Roundup applied (commonly used as glyphosate equivalent). The survival of the embryo was at the lowest in the 20 mg/L glyphosate group. Glyphosate-exposed embryos did not hatch timely on day 8 except for the 0.5 mg/L group. Among those that had delayed hatching, embryos from the two highest glyphosate groups showed a significant delay, which exceeded day 14 after fertilization (Figure 1).

### 3.2. Development and Growth

**Figure 2:**
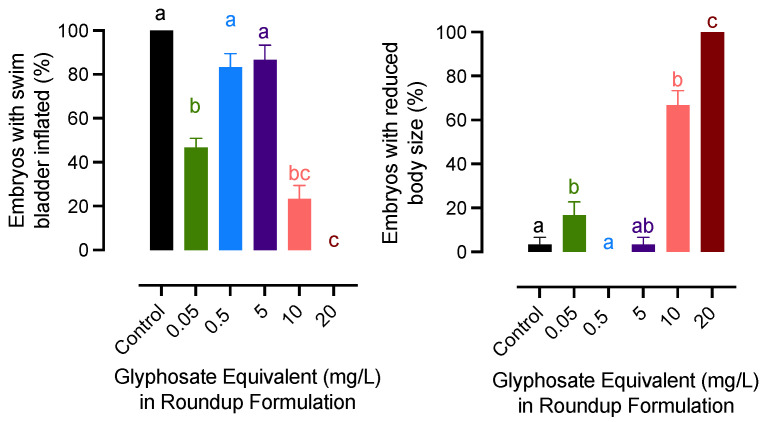
Effects of exposure to glyphosate equivalent in Roundup formulation on swim bladder inflation and growth rate of newly hatched medaka larvae. Bars with different letters on them indicate that treatments groups they represent are significantly different (*p* < 0.05) from each other. Data represent mean with an standard error of the mean.

The lowest concentration (0.05 mg/L) and the highest concentration groups had similar effects on embryonic development and growth. A significant decrease in swim bladder inflation was recorded for embryos exposed to 0.05, 10, and 20 mg/L of glyphosate groups. A similar pattern was observed for the growth of newly hatched embryos. The body size was significantly decreased in embryos exposed to 0.05, 10, and 20 mg/L of glyphosate, while the teratogenic effects were not significant in fry from 0.5 to 5 mg/L groups (Figure 2).

### 3.3. Expression of Thyroid Hormone Receptors

**Figure 3:**
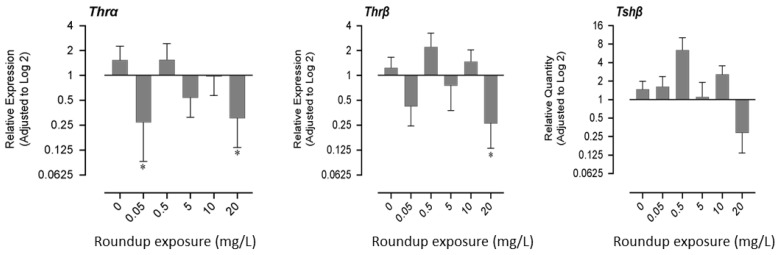
Effects of exposure to glyphosate equivalent in Roundup formulation on the expression of thyroid hormone receptors (α,β) and thyroid stimulating hormone-β.3.4. Expression of hatching enzyme protease, Acetylcholinesterase, and DNA methyltransferase-1. The asterisk indicates that the treatment group is significantly different (*p* < 0.05) from the control group.

Exposure to very low (0.05 mg/L) and very high concentrations (20 mg/L) of glyphosate had similar effects on gene expression patterns. mRNA expression of thyroid receptor α decreased significantly in fry exposed to 0.05 mg/L and 20 mg/L of glyphosate, while the mRNA expression of thyroid receptor β decreased significantly only in the 20 mg/L exposure group. mRNA profile of thyroid stimulating hormone (*tshβ*) showed a non-monotonic response; it increased in lower exposure concentrations and decreased in higher exposed concentrations (Figure 3).

**Figure 4:**
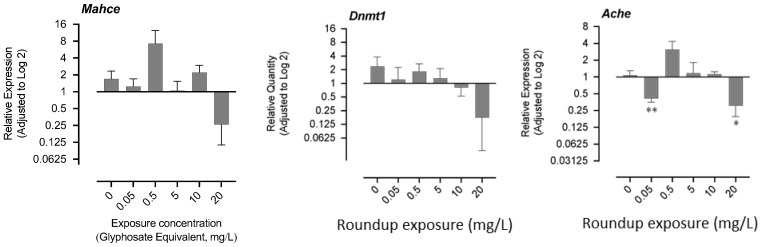
Effects of exposure to glyphosate equivalent in Roundup formulation on expression of acetylcholinesterase and DNA methyltransferase-1. The asterisk indicates that the treatment group is significantly different (* *p* < 0.05, ** *p* < 0.01)) from the control group.

The mRNA levels of hatching enzyme-like protease gene (*mahce*), acetylcholinesterase (*ache*), and DNA methyltransferase-1 (*dnmt1*) are shown in Figure 4. The exposure to 0.05 mg/L and 20 mg/L resulted in a significant decrease in the mRNA level of *ache*, while a nonsignificant increase was recorded in the exposure group of 0.5 mg/L (Figure 4). There was no significant change in the mRNA levels of *mahce* and *dnmt1,* although the trend showed an apparent decrease in the 20 mg/L group, where most of the fry could not hatch (Figure 4).

### 3.4. Expression of Antioxidants and Apoptosis-Related Genes

mRNA expression of antioxidant genes such as catalase, glutathione-S-transferase, and superoxide dismutase-1 decreased in all the groups exposed to glyphosate. However, the decrease was not statistically significant. Expression of apoptotic genes like caspase-3 increased in the 0.05 mg/L exposure group, but the increase was not statistically significant (Supplementary Figure S1).

## 4. Discussion

Glyphosate is the active ingredient of herbicides widely used in the United States and Europe [18,19]. The most commonly used glyphosate-based herbicide is Roundup which contains isopropylamine, a salt of glyphosate, as an active ingredient. The concentration of glyphosate in Roundup and other formulations is expressed as glyphosate acid equivalent. Although glyphosate targets plants through the Shikimic acid pathway, it is now evident that glyphosate also affects non-targeted species, most predominantly fish species. Most studies focusing on the adverse effects of glyphosate used relatively high concentrations; therefore, it remains unknown whether environmentally relevant or lower concentrations cause any effects in the non-targeted species. The present study focused on the effects of waterborne exposure to environmentally relevant and lower concentrations of glyphosate. We present here that the effects of low concentrations (0.05 mg/L) of Roundup are similar to those of high concentrations (20 mg/L, ~LC_50_). We also present that low concentrations of Roundup can impair molecular pathways associated with thyroid hormone function in fish, which suggests potential long-term effects on growth and metabolism.

Glyphosate in the environment is found in low concentrations. It is not clearly understood if such concentrations are detrimental to fish larvae inhabiting glyphosate-contaminated waters. Our data reported for the first time that low concentrations (0.05 mg/L) of Roundup had almost similar effects to high concentrations (20 mg/L). This study also reported that extremely low concentrations of Roundup impair the thyroid function in fish, which can lead to long-term effects on growth and metabolism.

Analysis of mRNA levels of genes involved in the thyroid pathway is a promising tool for studying the effect of environmental contaminants on the early developmental stages of organisms. The expression profile of genes of the thyroid pathway can be used to correlate with the circulating levels of thyroid hormones [9,20,21,22]. In the present study, we observed the expression profile of genes (*thrα*, *thrβ,* and *tshβ*) involved in the HPT axis. Exposure to 0.05 and 20 mg/L of Roundup resulted in a significant decrease in thyroid hormone receptors (*thrα* and *thrβ*), while the expression of *tshβ* only decreased with 20 mg/L exposure. The observed alterations in thyroid hormone-related genes clearly suggest that Roundup formulation containing the specified amount of glyphosate acid equivalent can be harmful for fish larvae and probably for their growth. Glyphosate has been reported to influence the hypothalamus–pituitary–thyroid (HPT) axis in mammals. However, the data is not consistent. In a previous study with amphibians, it was reported that glyphosate alone did not affect the HPT axis, the toxicity observed in Roundup formulations was due to the surfactant POEA [10]. On the other hand, studies with mice and rats reported that exposure to glyphosate influenced the genes involved in the HPT axis leading to altered circulating thyroid hormone levels [11,23,24]. A significant correlation was reported between urinary glyphosate and increased T4 levels in farmers spraying glyphosate-based herbicides in the United States [25].

A decrease in hatching rate and delayed hatching was observed in medaka embryos exposed to 0.05 and 20 mg/L of Roundup. Decreased survival and hatching may be linked to the disruption of the thyroid axis. Studies have shown that thyroid hormone plays a significant role in fish embryogenesis, larval development, growth, behavior, metabolism, osmoregulation, and reproduction [26,27,28,29,30,31]. In fish, thyroid hormones contribute largely to the transformation from the larval stage to the juvenile stage [32]. Environmental contaminants that can influence the development, hatching, and growth can lead to reduced fitness and even decreased population size in the natural environment. Roundup, even at very low exposure concentrations, significantly lowers the hatching rate and growth in the medaka larvae. A previous study from our laboratory reported that environmentally relevant (0.5 mg/L) concentrations of glyphosate and Roundup formulation exposure resulted in developmental defects, decreased hatching success, and altered the expression profile of *dnmt1*, *dnmt3aa*, *tet1*, *tet2*, and *tet3* [16], suggesting that lower concentrations of glyphosate and Roundup may have teratogenic effects in developing larvae. The expression of molecular markers for oxidative stress and cell death did not show significant alterations, suggesting that such effects might have been mediated via endocrine disruption, such as thyroid hormone pathways.

Acetylcholinesterase (ACHE) is an important enzyme that degrades neurotransmitters, acetylcholine. A decreased activity of ACHE resulted in the accumulation of acetylcholine in the synapse, leading to hyperactivation of the postsynaptic membrane that may result in the death of the organism [33]. In addition to its enzymatic function, ACHE has been found to influence muscle and neural development in zebrafish [34]. Exposure to 0.05 and 20 mg/L of Roundup resulted in a significant reduction in *ache* mRNA expression. A similar decrease was observed in *Cnesterodon decemmaculatus* exposed to 1 mg/L of glyphosate, confirming that glyphosate can influence acetylcholinesterase expression at various exposure concentrations. Lower concentrations (250 µg/L) of pure and commercial glyphosate resulted in a significant decrease in acetylcholinesterase activity in zebrafish, which may lead to abnormal feeding behavior [35]. The reason for larval mortality could be linked to the inability of the embryo to swim correctly and eat the food provided. Additionally, the inability to hatch from the egg could also be attributed to the inefficient movement of the fry within the chorion to break it on day 8.

The present results showed an interesting trend in overall dose response to Roundup exposure. The lower concentration (0.05 mg/L) tested yielded similar results as the higher exposure concentration (20 mg/L) of Roundup. Similar observations were reported by Farina et al. [8] Lower and higher concentrations of glyphosate (0.3 and 3 µg/L) influenced the behavior of zebrafish due to decreased levels of neurotransmitters like dopamine and norepinephrine [8], suggesting that high experimental concentrations have similar effects on behavior as the lower experimental concentration tested. One plausible explanation for this observation could be the ability of embryos to receive low concentrations of Roundup as an endogenous signal, but untimely, to divert their endocrine pathways towards developmental abnormalities, whereas higher concentrations of Roundup could be exerting toxic effects directly affecting the viability of cells in the developing embryos.

## 5. Conclusions

In conclusion, this study provides insights into the teratogenic effects of Roundup in fish. It provides evidence that Roundup can have deleterious effects on the development and survival of fish embryos, not only at pharmacologically toxic levels but also at environmentally relevant levels. The concentrations of Roundup below the levels of environmental relevance can alter development and influence the genes involved in thyroid hormone synthesis and binding, which can be attributed to decreased growth and survival.

## Data Availability

Data is contained within the article. The data presented in this study are available on request from the corresponding author.

## Supplementary Materials

The following supporting information can be downloaded at: https://www.mdpi.com/article/10.3390/jox13030032/s1, Reference [36] are cited in the supplementary materials. Figure S1: Expression of genes encoding *bax1*, *caspase3*, *catalase*, *gst*, and *sod1* in the whole larvae of medaka exposure to various concentrations of glyphosate in commercially available Roundup formulation. Table S1: The sequences of the primers used in the present study.
