# Supplementary material for: Effects of Chronic Roundup Exposure on Medaka Larvae
Source: J Xenobiot. 2023 Sep 14;13(3):500–8. doi: 10.3390/jox13030032 (PMC10533122; doi:10.3390/jox13030032)
Supplement: Supplementary file 1 [file jox-13-00032-s001.zip › jox-2511522-supplementary.pdf]

Supplementary Information

Effects of Chronic Roundup Exposure on Medaka Larvae

Deborah Killian [1], Mehwish Faheem [1,2], Beh Reh [1], Xuegeng Wang [1,3] and Ramji Kumar Bhandari [1,2,*]

1 Department of Biology, University of North Carolina Greensboro, Greensboro, NC 27412, USA; wangxuegeng@scnu.edu.cn (X.W.)
2 Division of Biological Sciences, University of Missouri, Columbia, MO 65211, USA
3 Institute of Modern Aquaculture Science and Engineering, College of Life Sciences, South China Normal University, Guangzhou 510631, China
* Correspondence: bhandarir@missouri.edu

Table S1. The sequences of the primers used in the present study. Primers were designed using the https://useast.ensembl.org/index.html and Primer3 software [36].

	Sequence	
Primer Name	Forward	Reverse
ef1a	AGAAGGAAGCCGCTGAGATGG	GCTCAGCCTTCAGTTTGTCCAA
thr	AGGTGGATCTGGAAGCCTTC	TCACAAGGCAGCTCAGAGAA
thr	TGAGGGTTGCAAGGGATTCT	CGCACTTCCCCTCGTACTTA
tsh	CTCAAGGGACAGCAACATGAG	TTGGACTCAAGGGGACAGC
thr a	CATGTTTTCAGGGCTGCCTT	CTCGCTTTCTGGATCGTAGC
ache	CTAGCAGGGGTCAAGATGGG	TGTTCTCGTCCATCCAGTCC
mahce	AAGAAAATGGTCATGAAGCCCA	GTCTTAGGGGCCAGCAGATC
dnmt1	CTTCTGCCGCTGGAAAGAC	CCTCCTATCGGCGACCTG
bax	CTCAGAGGGTACGTGATTGAA	CAGCTGCTCCACCACTTCTT
caspase3	TCAGCCAAATCCCAGGTCTA	AGGGTCGGCGTCTTGTTTTA
catalase	GCGGTACAACAGCGCAGATGAAG	GGATGGACGGCCTTCAAGTTC
gst	CACGGCCATCCTGAAATACCT	GTCAGCCGGGAACCAATGAT
sod1	GGCACTCTGTATGCCGGCTGC	GTTACCAAAGTCTCCCGGATG

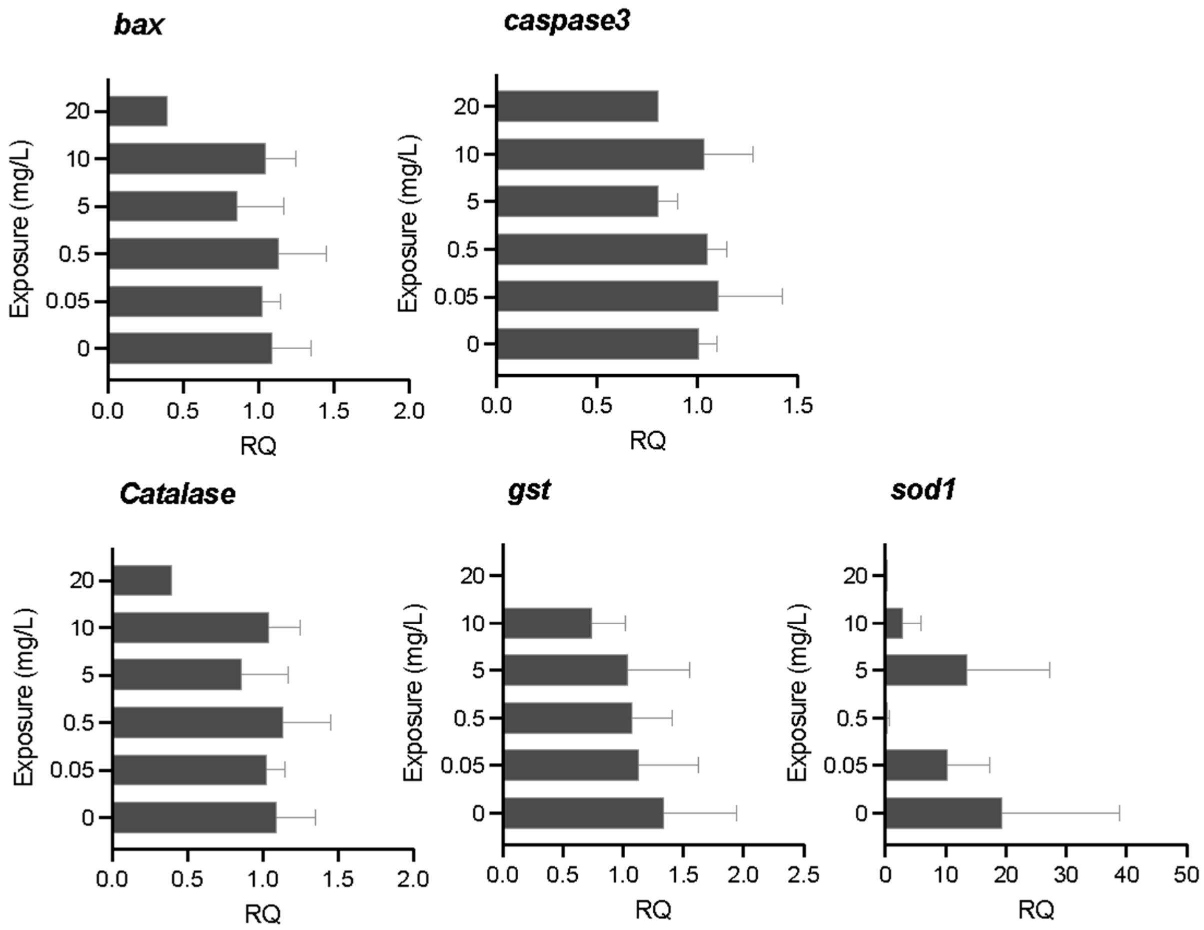

Figure S1. Expression of genes encoding *bax1, caspase3, catalase, gst,* and *sod1* in the whole larvae of medaka exposure to various concentrations of glyphosate in commercially available Roundup formulation. The mRNA levels were determined by real-time quantitative PCR using the primers shown in Table S1. No statistical significance was observed, although effects tended to be profound in larvae exposed to 20 mg/L of glyphosate.
